# Supplementary material for: Identification of the Inner Cell Mass and the Trophectoderm Responses after an In Vitro Exposure to Glucose and Insulin during the Preimplantation Period in the Rabbit Embryo
Source: Cells. 2022 Nov 25;11(23):3766. doi: 10.3390/cells11233766 (PMC9736044; doi:10.3390/cells11233766)
Supplement: Supplementary file 1 [file cells-11-03766-s001.zip › Supplementary Figures_nov4.pptx]

## Slide 1
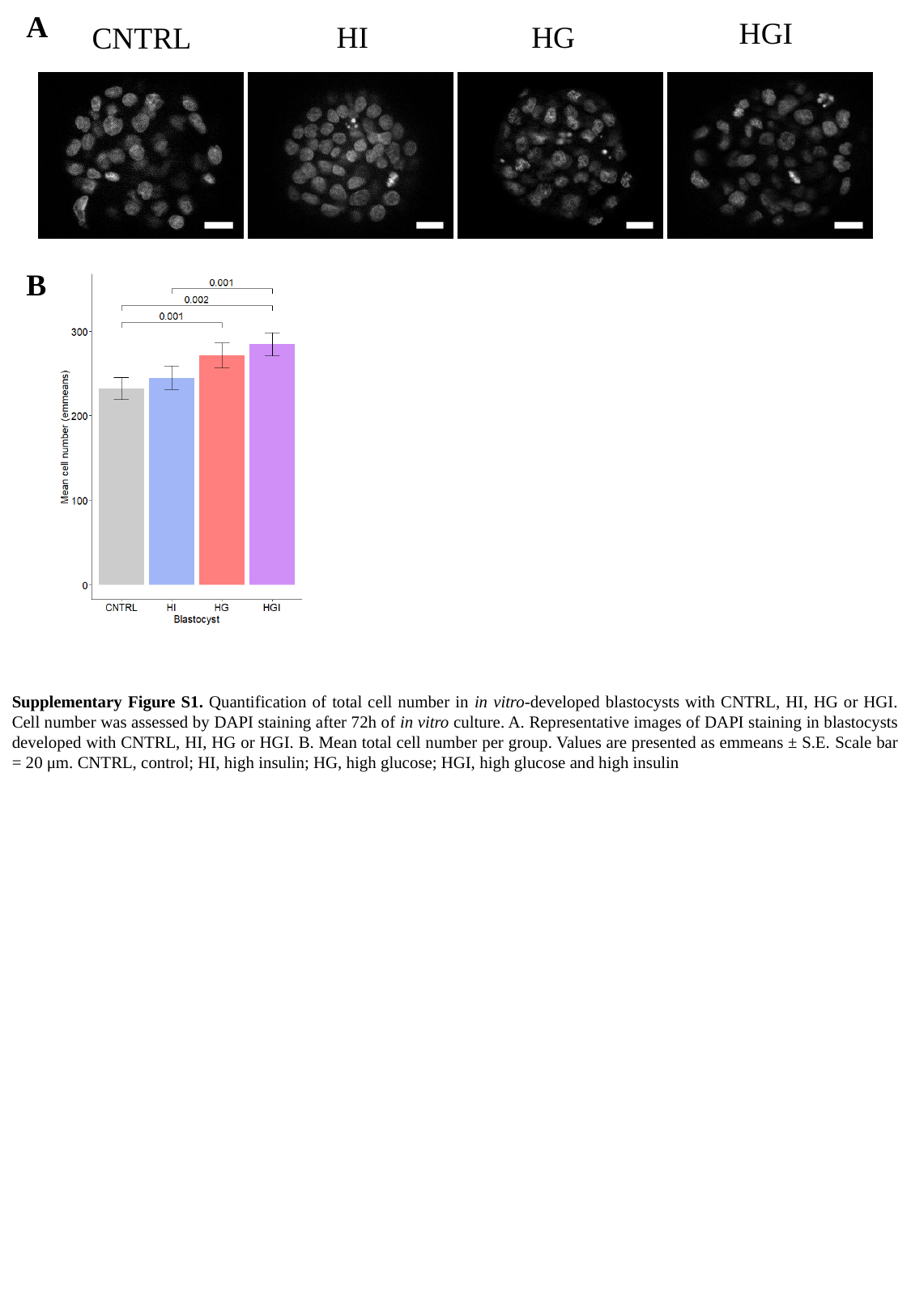

A
HGI
HI
HG
CNTRL
B
Supplementary Figure S1. Quantification of total cell number in in vitro-developed blastocysts with CNTRL, HI, HG or HGI. Cell number was assessed by DAPI staining after 72h of in vitro culture. A. Representative images of DAPI staining in blastocysts developed with CNTRL, HI, HG or HGI. B. Mean total cell number per group. Values are presented as emmeans ± S.E. Scale bar = 20 μm. CNTRL, control; HI, high insulin; HG, high glucose; HGI, high glucose and high insulin

## Slide 2
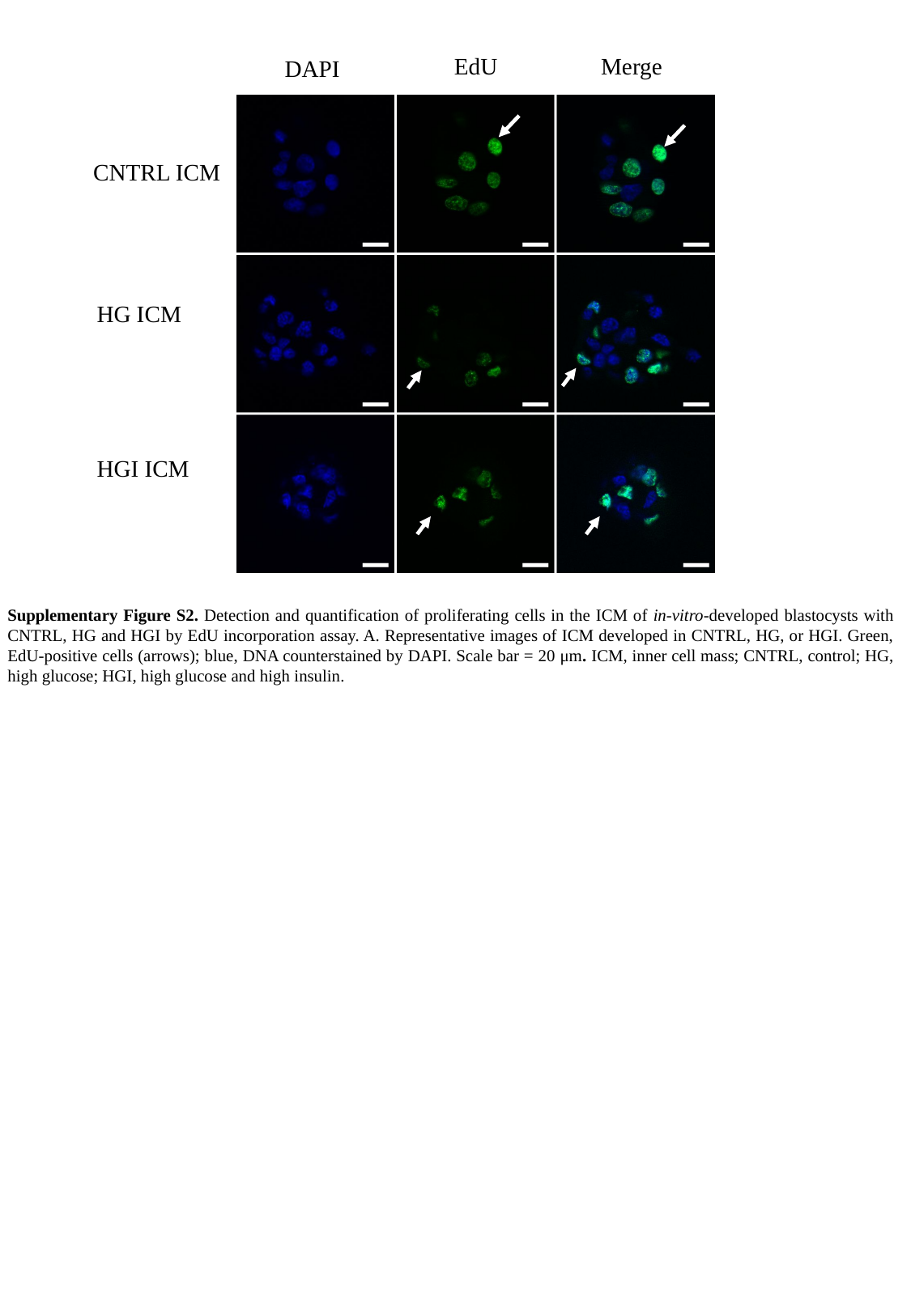

Merge
EdU
DAPI
CNTRL ICM
HG ICM
HGI ICM
Supplementary Figure S2. Detection and quantification of proliferating cells in the ICM of in-vitro-developed blastocysts with CNTRL, HG and HGI by EdU incorporation assay. A. Representative images of ICM developed in CNTRL, HG, or HGI. Green, EdU-positive cells (arrows); blue, DNA counterstained by DAPI. Scale bar = 20 μm. ICM, inner cell mass; CNTRL, control; HG, high glucose; HGI, high glucose and high insulin.

## Slide 3
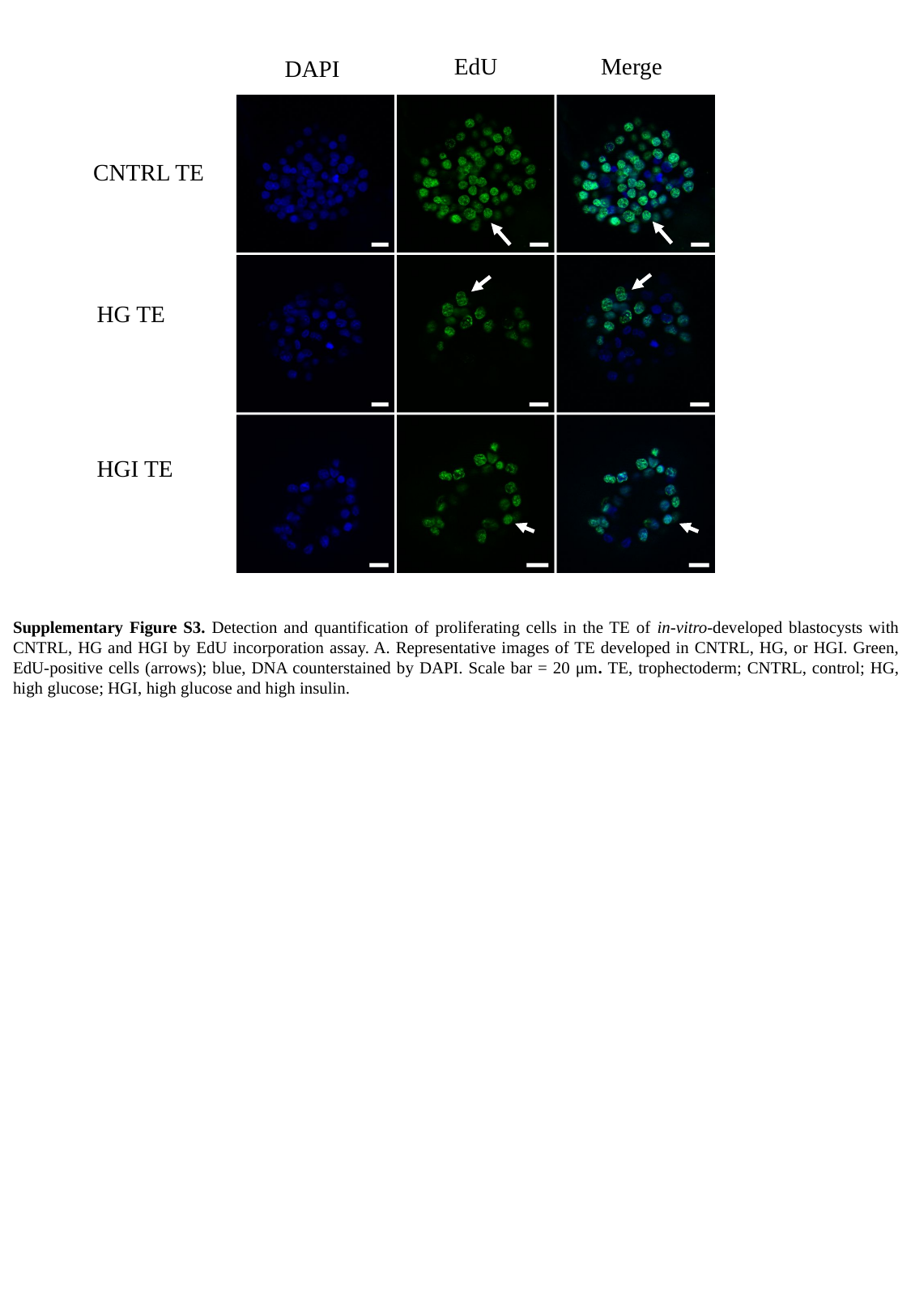

Merge
EdU
DAPI
CNTRL TE
HG TE
HGI TE
Supplementary Figure S3. Detection and quantification of proliferating cells in the TE of in-vitro-developed blastocysts with CNTRL, HG and HGI by EdU incorporation assay. A. Representative images of TE developed in CNTRL, HG, or HGI. Green, EdU-positive cells (arrows); blue, DNA counterstained by DAPI. Scale bar = 20 μm. TE, trophectoderm; CNTRL, control; HG, high glucose; HGI, high glucose and high insulin.

## Slide 4
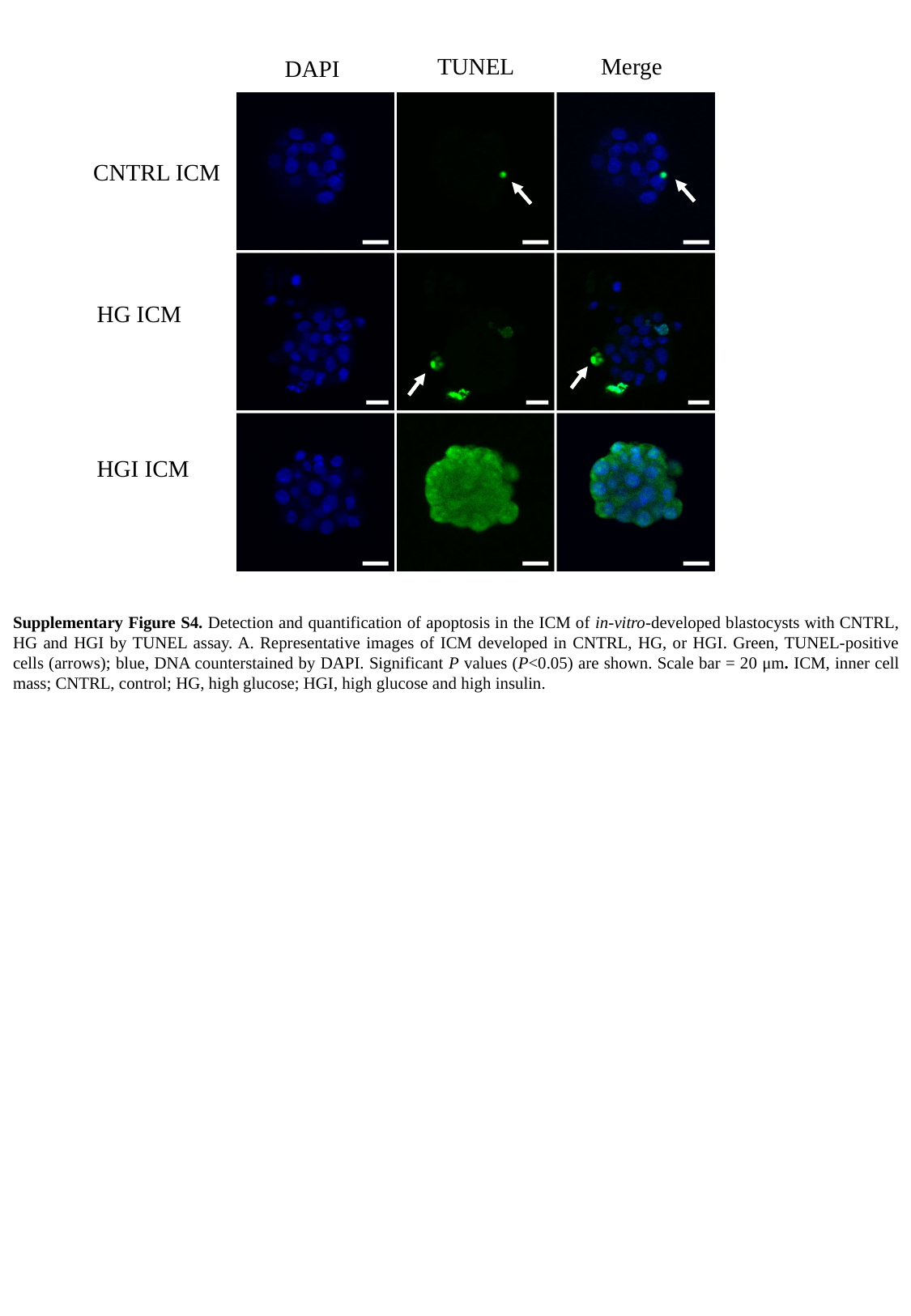

Merge
TUNEL
DAPI
CNTRL ICM
HG ICM
HGI ICM
Supplementary Figure S4. Detection and quantification of apoptosis in the ICM of in-vitro-developed blastocysts with CNTRL, HG and HGI by TUNEL assay. A. Representative images of ICM developed in CNTRL, HG, or HGI. Green, TUNEL-positive cells (arrows); blue, DNA counterstained by DAPI. Significant P values (P<0.05) are shown. Scale bar = 20 μm. ICM, inner cell mass; CNTRL, control; HG, high glucose; HGI, high glucose and high insulin.

## Slide 5
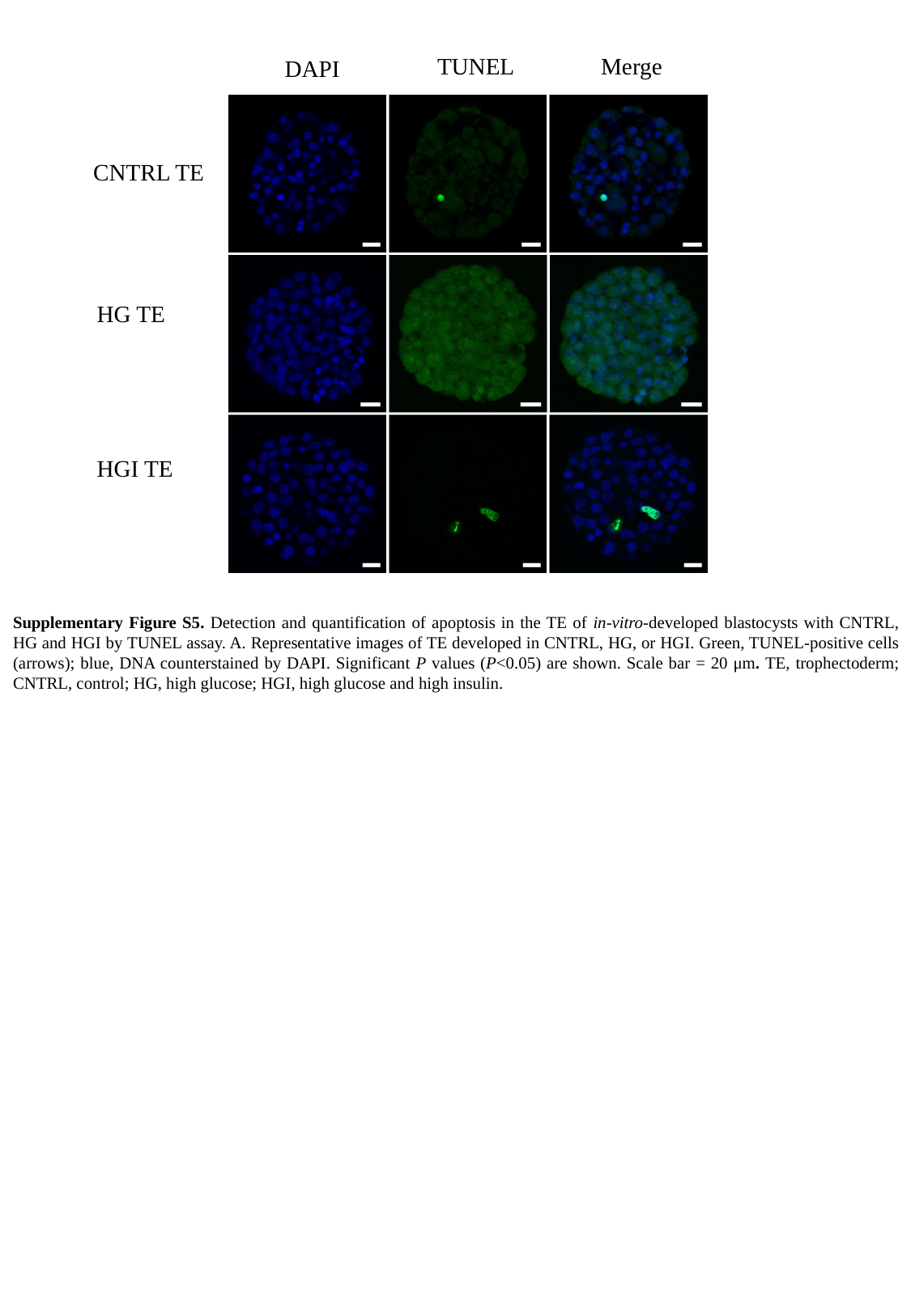

Merge
TUNEL
DAPI
CNTRL TE
HG TE
HGI TE
Supplementary Figure S5. Detection and quantification of apoptosis in the TE of in-vitro-developed blastocysts with CNTRL, HG and HGI by TUNEL assay. A. Representative images of TE developed in CNTRL, HG, or HGI. Green, TUNEL-positive cells (arrows); blue, DNA counterstained by DAPI. Significant P values (P<0.05) are shown. Scale bar = 20 μm. TE, trophectoderm; CNTRL, control; HG, high glucose; HGI, high glucose and high insulin.

## Slide 6
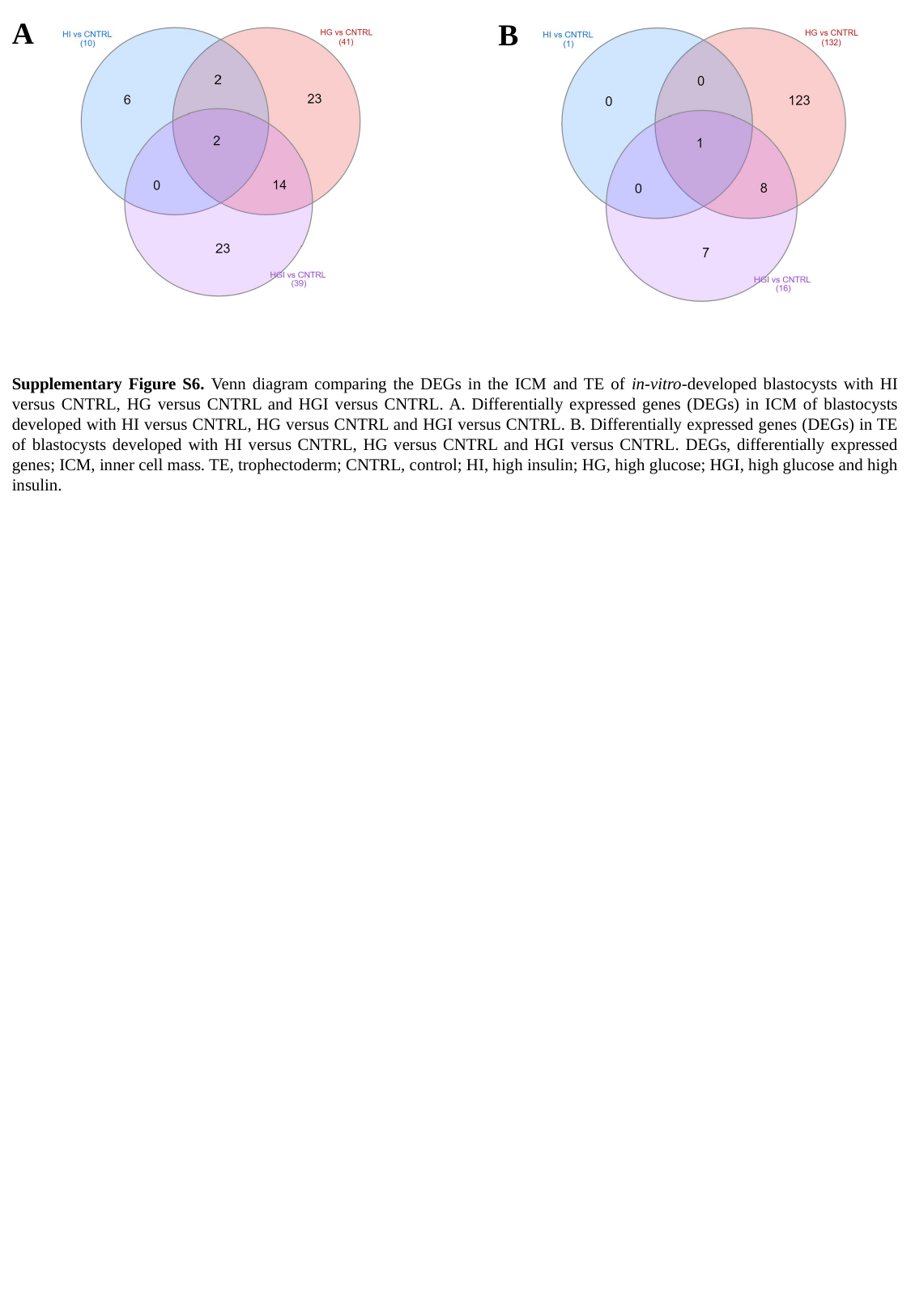

A
B
Supplementary Figure S6. Venn diagram comparing the DEGs in the ICM and TE of in-vitro-developed blastocysts with HI versus CNTRL, HG versus CNTRL and HGI versus CNTRL. A. Differentially expressed genes (DEGs) in ICM of blastocysts developed with HI versus CNTRL, HG versus CNTRL and HGI versus CNTRL. B. Differentially expressed genes (DEGs) in TE of blastocysts developed with HI versus CNTRL, HG versus CNTRL and HGI versus CNTRL. DEGs, differentially expressed genes; ICM, inner cell mass. TE, trophectoderm; CNTRL, control; HI, high insulin; HG, high glucose; HGI, high glucose and high insulin.
